# circ-miR-524 accelerates the growth of liver cancer cells by inducing DNA damage repair through K-RAS

**DOI:** 10.1016/j.gendis.2025.101675

**Published:** 2025-05-08

**Authors:** Yanan Lu, Dongdong Lu

**Affiliations:** a School of Life Science, Jiangsu University, Zhenjiang, Jiangsu 212013, China; b School of Life Science and Technology, Tongji University, Shanghai 200092, China

Circular RNAs (circRNAs) are endogenous RNAs formed by back-splicing^1^ and possess a more stable molecular structure.^2^ circRHOT1 promotes HCC progression by inducing nuclear receptor subfamily 2 group F member 6 (NR2F6) expression^3^ and m^6^A-mediated up-regulation of circMDK and circSTX6 facilitates tumorigenesis.^4^ Wang et al revealed that circTGFBR2 was a novel tumor promoter circRNA and promoted HCC progression.^5^ However, the role and molecular mechanism of circ-miR-524 in hepatocarcinogenesis have been poorly elucidated. In this study, we clearly demonstrate that circ-miR-524 accelerates the growth of liver cancer cells by altering transcriptome, proteome, and DNA damage repair. In particular, circ-miR-524 enhances the expression of K-RAS, which determines the carcinogenic function of miR-circ-524. These results provide a basis for research on liver cancer prevention, diagnosis, and treatment.

To explore the effect of circ-miR-524 on liver cancer cells, we cloned the miR-524 (RefSeq: MI0003160) precursor sequence (UCUCAUGCUGUGACCCUACA AAGGGAAGCACUUUCUCUUGUCCAAAGGAAAAGAAGGCGCUUCCCUUUGGAGUGUUACGGUUUGAGA) into the lentiviral vector pLVX-circRNA-ZsGreen-Puro (pLVX-circ-miR-524) and prepared rLV-circ-miR-524 lentivirus. Next, rLV-circ and rLV-circ-miR-524 were used to infect liver cancer cells Hep3B (Fig. S1A). circ**-**miR-524 was overexpressed in the rLV-circ**-**miR-524 group compared with the rLV-circ group (Fig. 1A, B). The proliferation ability was significantly increased in the rLV-circ-miR-524 group compared with the rLV-circ group (24 h: *p* = 0.0067; 48 h: *p* = 0.0017; 72 h: *p* = 0.00434) (Fig. 1C). The cellular colony formation ability was significantly increased in the rLV-circ-miR-524 group compared with the rLV-circ group (19.45% ± 4.88% *vs.* 44.33% ± 10.26%; *p* = 0.0072) (Fig. 1D; Fig. S1B). The weight of transplanted tumors was significantly increased in the rLV-circ-miR-524 group compared with the rLV-circ group (0.143 ± 0.035 g *vs*. 0.902 ± 0.121 g; *p* = 0.000025) (Fig. 1E; Fig. S1C, D). As shown in Figure 1F, the well-differentiated cells were significantly decreased in the rLV-circ-miR-524 group compared with the rLV-circ group (27.52% ± 6.67% *vs*. 10.88% ± 2.36%; *p* = 0.00074). The poorly differentiated cells were significantly increased in the rLV-circ-miR-524 group compared with the rLV-circ group (14.51% ± 2.25% *vs*. 31.48% ± 5.39%; *p* = 0.00046). The expression of proliferating cell nuclear antigen (PCNA) was significantly increased in the rLV-circ-miR-524 group compared with the rLV-circ group (32.17% ± 3.45% *vs*. 70.19% ± 7.49%; *p* = 0.000024) (Fig. 1G; Fig. S1E).

**Figure 1:**
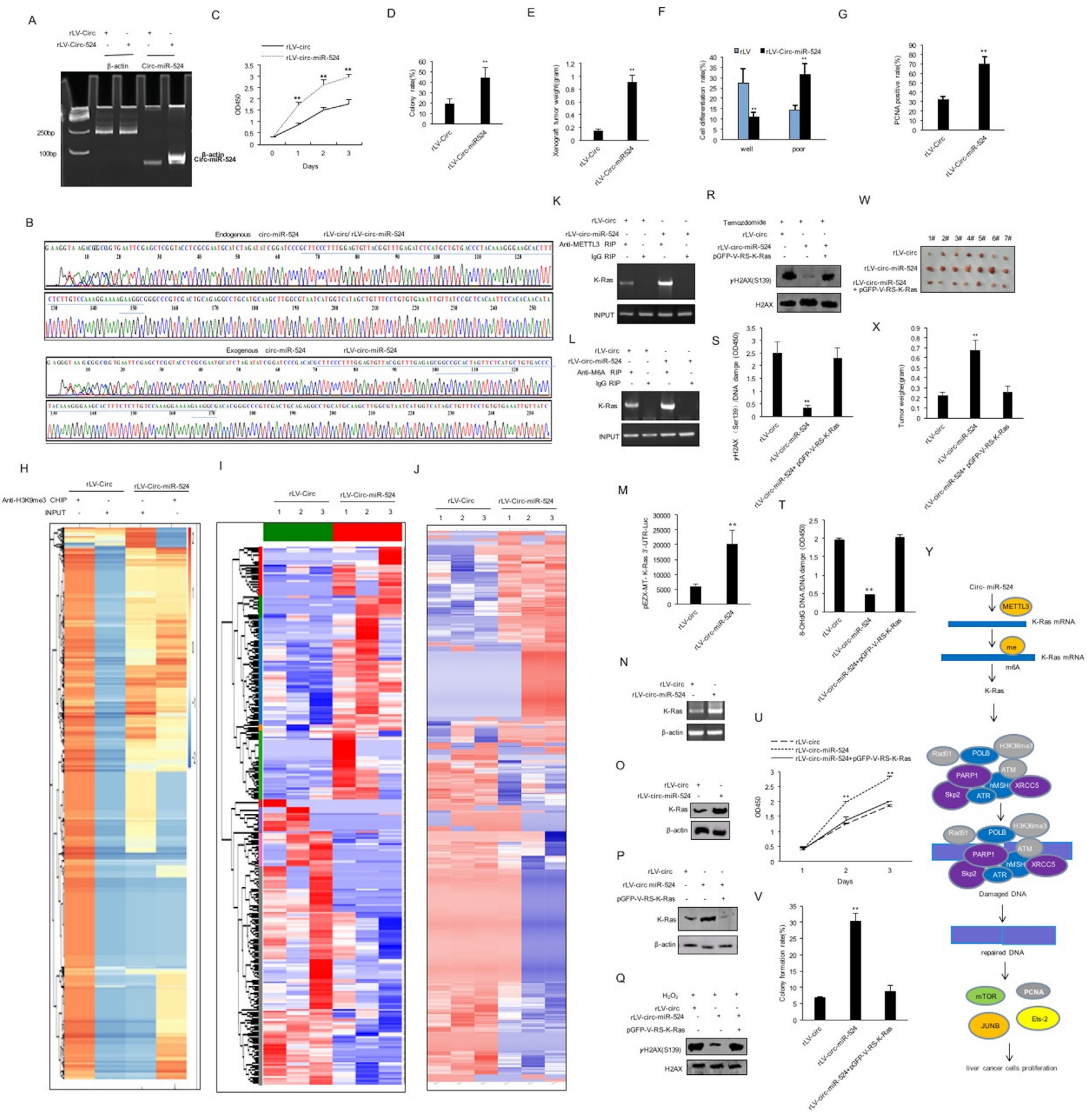
circ-miR-524 accelerates the growth of liver cancer cells by inducing DNA damage repair through K-RAS. **(A)** Hep3B cells were infected with rLV-circ-miR-524, and the circ-miR-524 was detected by back-to-back reverse transcription PCR. β-actin was used as the internal reference gene. **(B)** Sequencing for circ-miR-524. **(C)** The CCK8 method was used to determine the cell proliferation ability. The values of each group were expressed as mean ± standard deviation (SD) (*n* = 3). ∗∗*p* < 0.01 and ∗*p* < 0.05. **(D)** Analysis of colony-forming ability of cells. The values of each group were expressed as mean ± SD (*n* = 6). ∗∗*p* < 0.01 and ∗*p* < 0.05. **(E)** The xenograft tumor was dissected, and the tumor size (g) was subjected to comparison. The values of each group were expressed as mean ± SD (*n* = 6). ∗∗*p* < 0.01 and ∗*p* < 0.05. **(F)** Comparison of cell differentiation grade. The values of each group were expressed as mean ± SD (*n* = 6). ∗∗*p* < 0.01 and ∗*p* < 0.05. **(G)** Comparison of PCNA positive rate (%). The values of each group were expressed as mean ± SD (*n* = 6). ∗∗*p* < 0.01 and ∗*p* < 0.05. **(H)** Chromatin immunoprecipitation sequencing with anti-H3K9me3 high-throughput analysis was performed in human liver cancer cells (hierarchical clustering analysis). **(I)** Heatmap analysis (cluster) of all gene expression in the two groups. circ-miR-524 affects the transcriptome of human liver cancer cells. **(J)** Differential protein cluster heatmap. circ-miR-524 alters proteomics in liver cancer. The vertical is the clustering of samples, and the horizontal is the clustering of proteins. **(K)** The total RNA was extracted and analyzed by RNA immunoprecipitation. The samples were precipitated with anti-METTL3, and the immunoprecipitated RNA was analyzed by reverse transcriptase PCR with K-Ras primers. IgG RIP was used as the negative control. **(L)** The total RNA was extracted and analyzed by RNA immunoprecipitation. The samples were precipitated with anti-M6A, and the immunoprecipitated RNA was analyzed by reverse transcriptase PCR with K-Ras primers. IgG RIP was used as the negative control. **(M)** The assay of pEZX-MT-K-Ras-3′UTR-Luc activity. **(N)** The transcriptional ability of K-Ras was detected by reverse transcriptase PCR. β-actin was used as the internal reference gene. **(O)** The translational ability of K-Ras was detected by western blotting. β-actin was used as the internal reference gene. **(P)** The translation ability of K-Ras was detected by western blotting. β-actin was used as the internal reference gene. **(Q, R)** The expression of γH2AX (Ser139) was detected by western blotting. H2AX was used as the internal reference gene. **(S, T)** DNA damage assay (mean ± standard error of the mean; *n* = 3). ∗∗*p* < 0.01 and ∗*p* < 0.05. **(U)** The CCK8 method was used to determine the cell proliferation ability. The values of each group were expressed as mean ± SD (*n* = 6). ∗∗*p* < 0.01 and ∗*p* < 0.05. **(V)** The analysis of colony colony-forming ability of cells. The values of each group were expressed as mean ± SD (*n* = 3). ∗∗*p* < 0.01 and ∗*p* < 0.05. **(W)** The xenograft tumor was dissected. **(X)** Comparison of tumor size (g). The values of each group were expressed as mean ± SD (*n* = 7). ∗∗*p* < 0.01 and ∗*p* < 0.05. **(Y)** The schematic diagram of the molecular mechanism by which circ-miR-524 accelerates the growth of liver cancer cells by inducing DNA damage repair through K-RAS.

To explore how circ-miR-524 affected the epigenetic regulation of human live cancer cells Hep3B, chromatin immunoprecipitation sequencing with anti-H3K9me3 high-throughput analysis was performed. As shown in Figure 1H and Figure S2A–H, these results suggest that circ-miR-524 affects epigenetic regulation in human liver cancer cells. To study the effect of circ-miR-524 on the transcriptome of human liver cancer cells, the total RNA was extracted for RNA sequencing and detected by electrophoresis in the rLV-circ group and rLV-circ-miR-524 group. As shown in Figure 1I and Figure S3A–D, these results suggest that circ-miR-524 affects the transcriptome of human liver cancer cells. To study the effect of circ-miR-524 on the proteomics of human liver cancer cells, the total protein was extracted, and the proteolytic peptides were analyzed by label-free mass spectrometry in the rLV-circ group and rLV-circ-miR-524 group. As shown in Figure 1J and Figure S4A–E, these results suggest that circ-miR-524 affects the proteomics in liver cancer. Moreover, the binding ability of methyltransferase 3 (METTL3) to K-Ras mRNA (Fig. 1K) and the mRNA methylation modification of K-RAS (Fig. 1L), the K-Ras 3′UTR luciferase activity (Fig. 1M), and the expression of K-RAS (Fig. 1N, O) were significantly increased in the rLV-circ-miR-524 group compared with the rLV-circ group. As shown in Figure 1P and Figure S5A and B, circ-miR-524 was significantly increased in the rLV-circ-miR-524 group and the rLV-circ-miR-524 plus pGFP-V-RS-K-Ras group. K-Ras was significantly increased in the rLV-circ-miR-524 group and decreased in the rLV-circ-miR-524 plus pGFP-V-RS-K-Ras group compared with the rLV-circ group. Although the expression of γH2AX (the phosphorylated form of H2A.X variant histone (Ser139)) was significantly decreased in the rLV-circ-miR-524 group compared with the rLV-circ group, it was not significantly altered in the rLV-circ-miR-524 plus pGFP-V-RS-K-Ras group compared with the rLV-circ group (Fig. 1Q, R). Although the DNA damage repair ability was significantly increased in the rLV-circ-miR-524 group compared with the rLV-circ group, it was not significantly altered in the rLV-circ-miR-524 plus pGFP-V-RS-K-Ras group compared with the rLV group (Fig. 1S, T). Furthermore, although the expression of Ets-2, JUNB, PCNA, and mechanistic target of rapamycin (mTOR) was significantly decreased in the rLV-circ-miR-524 group compared with the rLV-circ group, it was not significantly altered in the rLV-circ-miR-524 plus pGFP-V-RS-K-Ras group and the rLV-circ-miR-524 plus rucaparib group compared with the rLV-circ group (Fig. S5C). Collectively, these observations suggest that circ-miR-524 increases the DNA damage repair ability dependent on K-Ras in human liver cancer cells. Moreover, although the proliferation ability was significantly increased in the rLV-circ miR-524 group compared with the rLV-circ group (24 h: *p* = 0.00054; 48 h: *p* = 0.00074), it was not significantly altered in the rLV-circ-miR-524 plus pGFP-V-RS-K-RAS group compared with the rLV-circ group (24 h: *p* = 0.141; 48 h: *p* = 0.072) (Fig. 1U). Although the colony formation ability was significantly increased in the rLV-circ-miR-524 group compared with the rLV-circ group (6.55% ± 0.69% *vs*. 30.11% ± 2.19%; *p* = 0.0029), it was not significantly altered in the rLV-circ-miR-524 plus pGFP-V-RS-K-RAS group compared with the rLV-circ group (6.55% ± 0.69% *vs*. 8.55% ± 1.95%; *p* = 0.0869) (Fig. 1V; Fig. S6A). Although the tumor weight was significantly increased in the rLV-circ-miR-524 group compared with the rLV-circ group (0.22 ± 0.04 g *vs.* 0.67 ± 0.11 g; *p* = 0.000021), it was not significantly altered in the rLV-Circ-miR-524 plus pGFP-V-RS-K-RAS group compared with the rLV-circ group (0.22 ± 0.04 g *vs.* 0.25 ± 0.06 g; *p* = 0.197) (Fig. 1W, X; Fig. S6B). Although the PCNA positive rate was significantly increased in the rLV-circ-miR-524 group compared with the rLV-circ group (41.06% ± 5.52% *vs.* 69.36% ± 7.37%; *p* = 0.00029), it was not significantly altered in the rLV-circ-miR-524 plus pGFP-V-RS-K-RAS group compared with the rLV-circ group (41.06% ± 5.52% *vs.* 5.78% ± 3.92%; *p* = 0.076) (Fig. S6C). Taken together, these observations suggest that K-RAS determines the carcinogenic function of miR-circ-524 in liver cancer.

In summary, the present study provides evidence for circ-miR-524 to play a role in hepatocarcinogenesis by altering transcriptome, proteome, and DNA damage repair. Anyhow, we demonstrate that abnormal expression of circ-miR-524 is very important in hepatocarcinogenesis. Our findings underscore the need for new approaches to further uncover the mechanisms underlying circ-miR-524-mediated functions in hepatocarcinogenesis.

## CRediT authorship contribution statement

**Yanan Lu:** Investigation. **Dongdong Lu:** Writing – review & editing, Writing – original draft, Visualization, Validation, Supervision, Software, Resources, Project administration, Methodology, Investigation, Funding acquisition, Formal analysis, Data curation, Conceptualization.

## Ethics declaration

All methods were carried out in accordance with the approved guidelines. All experimental protocols were approved by the Tongji University Institutional Committee. Informed consent was obtained from all subjects. The study was reviewed and approved by the China National Institutional Animal Care and Use Committee (ethics number: TJAB04222101).

## Conflict of interests

The authors declared no competing interests.
