# Supplementary material for: circ-miR-524 accelerates the growth of liver cancer cells by inducing DNA damage repair through K-RAS
Source: Genes Dis. 2025 May 8;12(6):101675. doi: 10.1016/j.gendis.2025.101675 (PMC12310062; doi:10.1016/j.gendis.2025.101675)
Supplement: Multimedia component 1 [file mmc1.docx]

**Supplemental Data**

**Keywords**

Circ-miR-524; liver cancer; transcriptomics; proteomics; DNA damage repair, K-RAS

**Materials and Methods**

**Cell Lines, Lentivirus** Human liver cancer cell line (Hep3B) was maintained in Dulbecco’s modified Eagle medium(Gibco) in a humidified atmosphere of 5% CO_2_ incubator at 37ºC. rLV-Circ, rLV-Circ-miR-524 were purchased from Wu Han viraltherapy Technologies Co. Ltd. pGFP-V-RS was purchased from Origene (Rockville, MD,USA) and pGFP-V-RS-K-RAS was constructed by ourselves.

**Western blotting** The proteins were separated on a 10% sodium dodecyl sulfate-polyacrylamide gel electrophoresis (SDS-PAGE) and transferred onto a nitrocellulose membrane. The blots were incubated with antibody at 4°C overnight. Following three washes, membranes were then incubated with secondary antibody at 4°C overnight. Signals were visualized by ECL system.

**Chromatin immunoprecipitation (CHIP) assay** Chromatin extracts were pre-cleared with Protein-A/G-Sepharose beads, and immunoprecipitated with specific antibody on Protein-A/G-Sepharose beads. After washing, elution and de-cross-linking, the ChIP DNA was detected by PCR).

**Cells proliferation CCK8 Assay** The cell proliferation reagent CCK8 is purchased from Roch and the operation according to the manufacturer instruction.

**Colony-Formation Efficiency Assay** Cells were plated on a six wells plate and were incubated at 37°C in humidified incubator for 7 days. Cell colonies were stained with 0. 05% Crystal Violet.

**Xenograft transplantation *in vivo*** Four-weeks male athymic Balb/C mice were injected with Hep3B cells at the armpit area subcutaneously. The mice were observed over 4 weeks, and then sacrificed to recover the tumors. The use of mice for this work was reviewed and approved by the institutional animal care and use committee in accordance with China national institutes of health guidelines.

**Mass spectrometric analysis** Mass spectrometric analysis of enzyme hydrolyzed peptides of protein without label free was performed according to according to the manufacturer operation manual (Shanghai Majorbio Bio-pharm Technology Co.,Ltd)

**Supplemental Figure and Figure Legends**

**
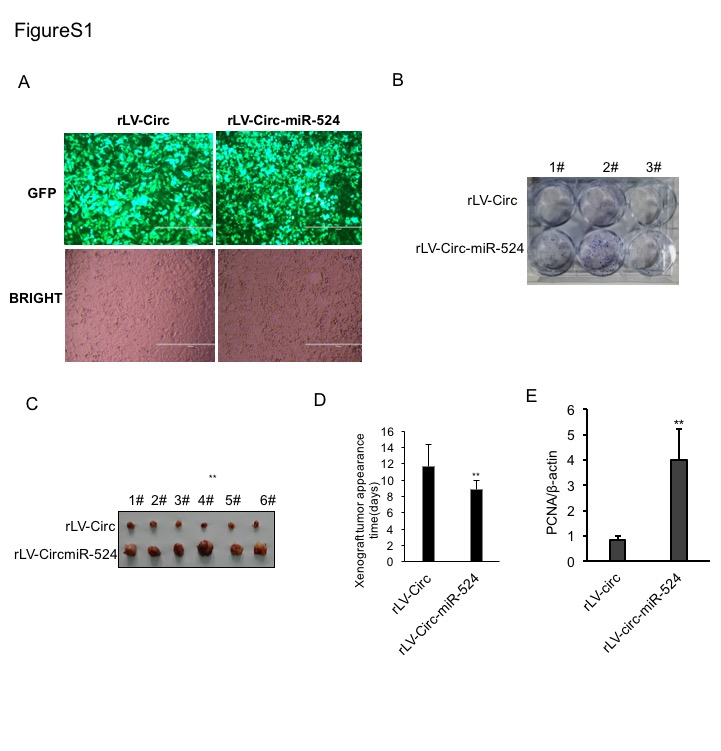
**

**FigureS1** Circ-miR-524 promotes the growth of liver cancer cells *in vitro and in vivo*. A. Hep3B cells were infected with rLV-Circ-miR-524 and the pictures were taken under fluorescence microscope. B. The colony forming ability of cells was measured. Photos of plate colonies. C. the xenograft tumor was dissected. D. Comparison of appearance time of xenograft tumor. The values of each group were expressed as mean ± SD (n = 6), * *, P < 0.01, and *, P < 0.05.E. The analysis of anti-PCNA immunohistochemical staining. The values of each group were expressed as mean ± SD (n = 6), * *, P < 0.01, and *, P < 0.05.


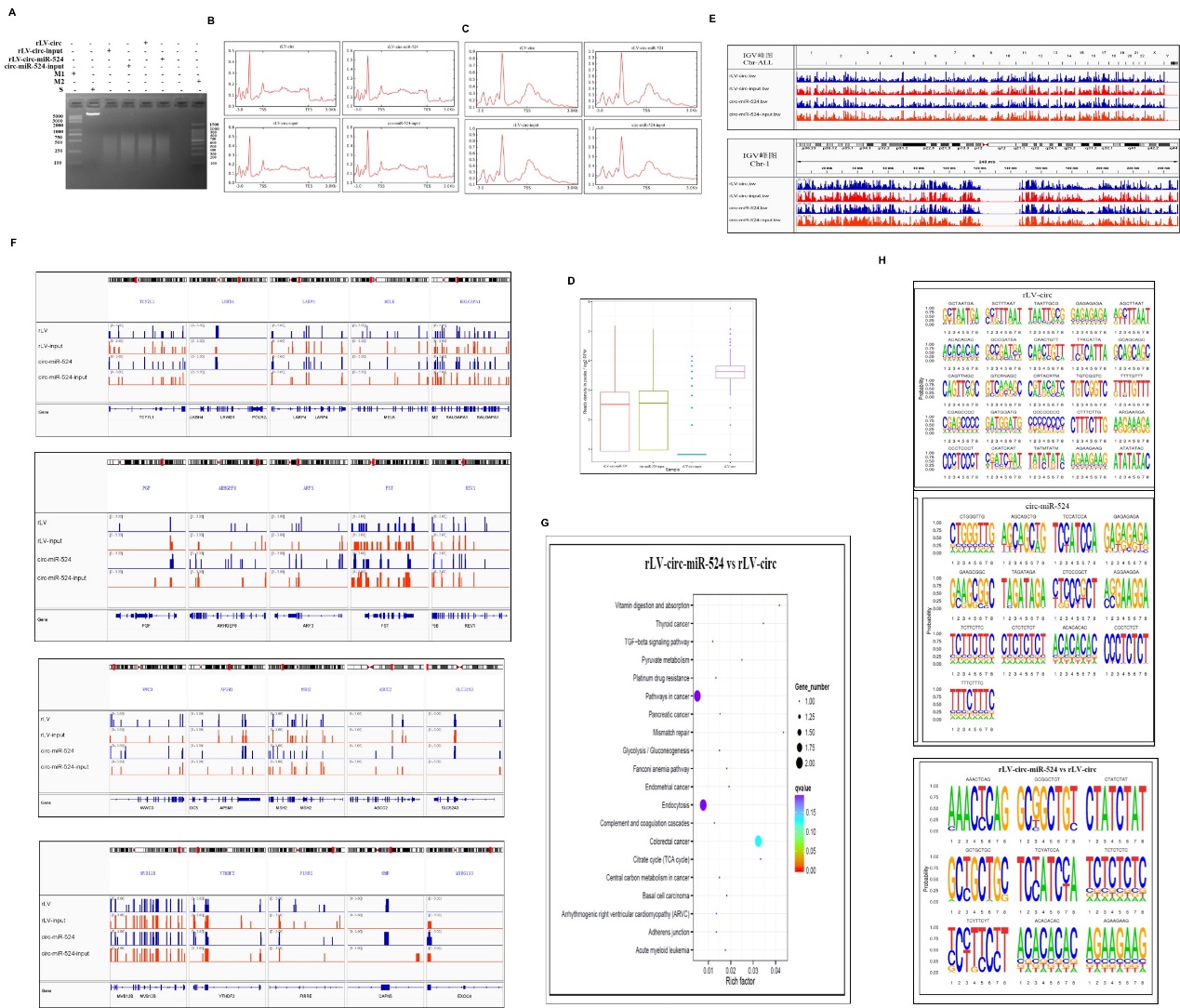


FigureS2 Chromatin immunoprecipitation sequencing (Chip-Seq) with anti-H3K9me3 high-throughput analysis was performed in human liver cancer cells. **A.**Cells were cross-linked by formaldehyde of 1% and DNA was extracted. Then the DNA fragments after ultrasonic fragmentation were identified by 1% agarose gel electrophoresis. B. **T**he distribution of reads relative gene positions in rLV-Circ group and rLV-Circ -miR-524 group. Abscissa: relative position of gene; Ordinate: reads density(RPKM). **C.** The average signal distribution on the 2KB region upstream and downstream of TSS in rLV group and rLV-miR-524 group. D. Boxplot plot of reads density distribution in peak region (RPM). **E.** IGV browser interface (Demo): visualization of the reads of the modification distribution of H3K9me3 on 23 pairs of chromosomes in rLV group and rLV-miR-524 group. F. IGV browser interface (Demo): visualization of the reads of modification distribution of H3K9me3 on gene region. G. KEGG Enrichment analysis of gene with peak difference.H.Motif sequence.


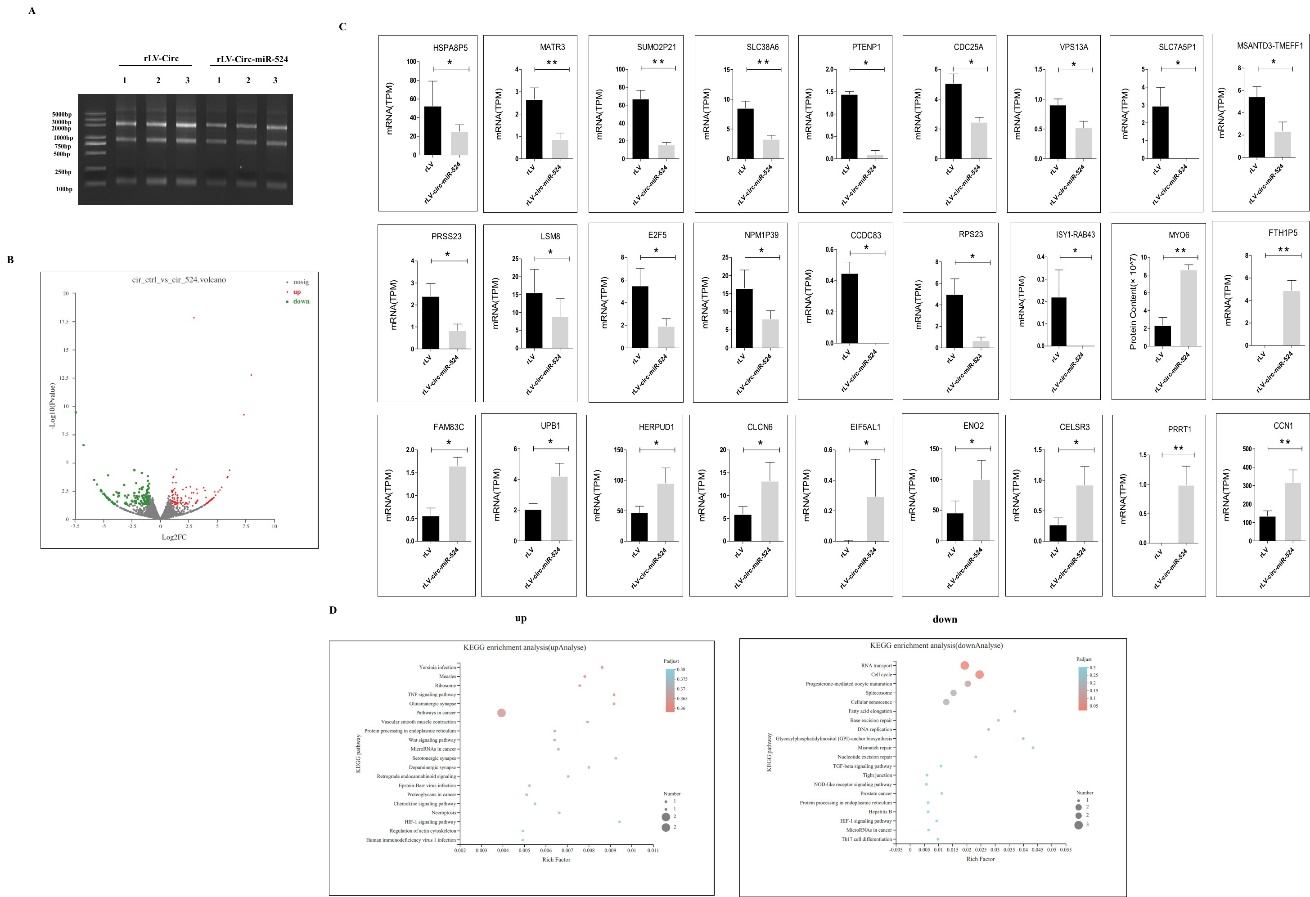


**FigureS3** miR-524 affects the transcriptome of human liver cancer cells. A. Total RNA was extracted and detected by 1% agarose gel electrophoresis. B. The volcanic map can visually show the distribution of different genes in each comparison combination. The abscissa represents the change of gene expression multiple (log2foldchange) and the ordinate represents the significant level of gene expression difference. The up-regulated genes are indicated by red dots and the down-regulated genes are indicated by green dots. C. down-regulated genes and up-regulated genes.D. The KEGG enrichment bubble and histogram diagram.


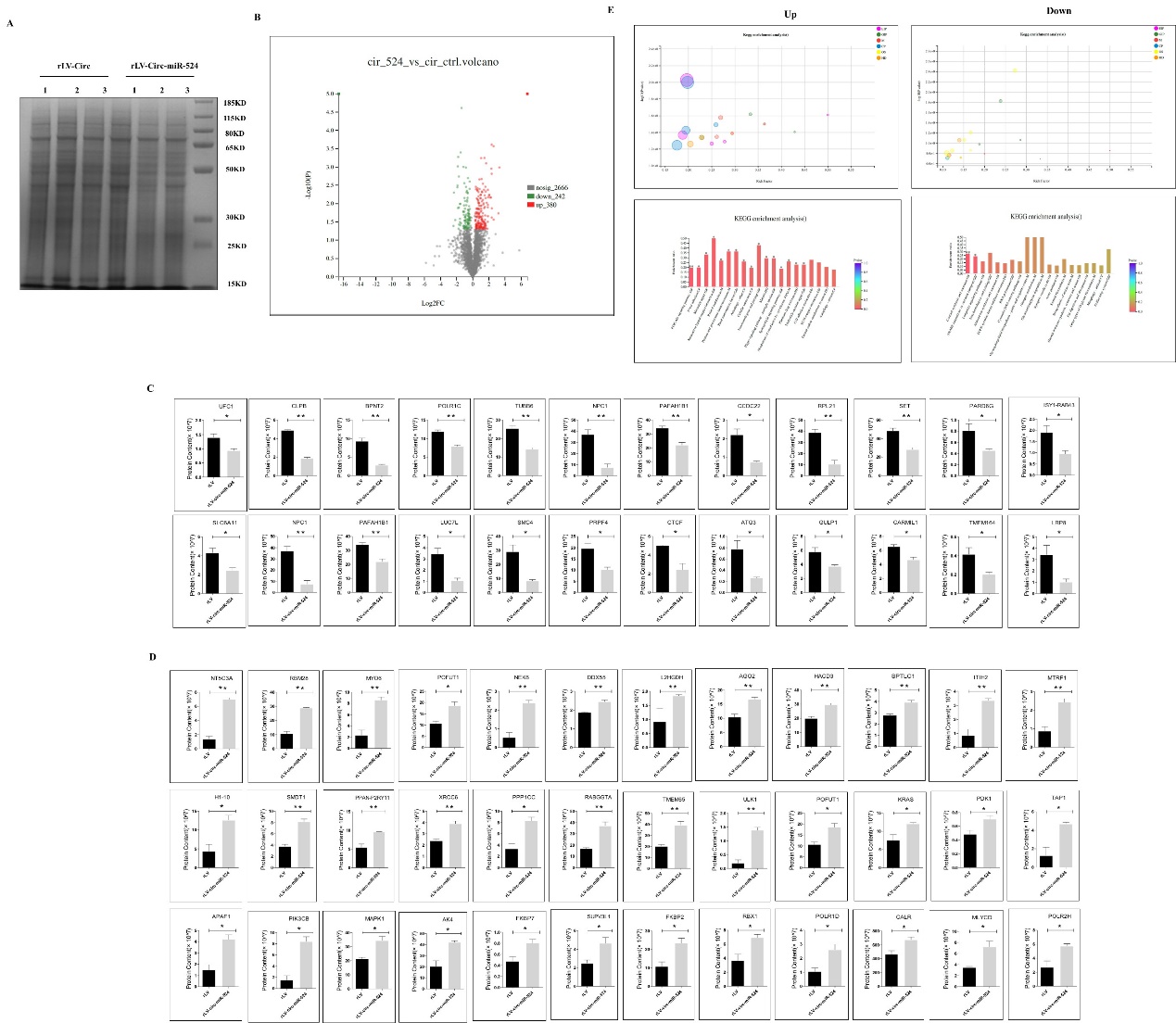


**Figure S4 Circ-miR-524 alters proteomics in liver cancer** A. The total protein was extracted and and analyzed by 10% SDS-PAGE electrophoresis. B. Differential protein volcano map . the abscissa represents the difference multiple (log2 value) of the differential protein, and the vertical axis represents pvalue (- log10 value). Black represents the protein with no significant difference, red represents the up-regulated protein, and green represents the down-regulated protein. C. Histogram of down-regulated proteins . D. Histogram of up-regulated proteins . E. The KEGG enrichment bubble and histogram diagram. The abscissa in the KEGG enrichment bubble diagram is the ratio of the number of differential proteins in the corresponding pathway to the number of total proteins identified in the pathway.


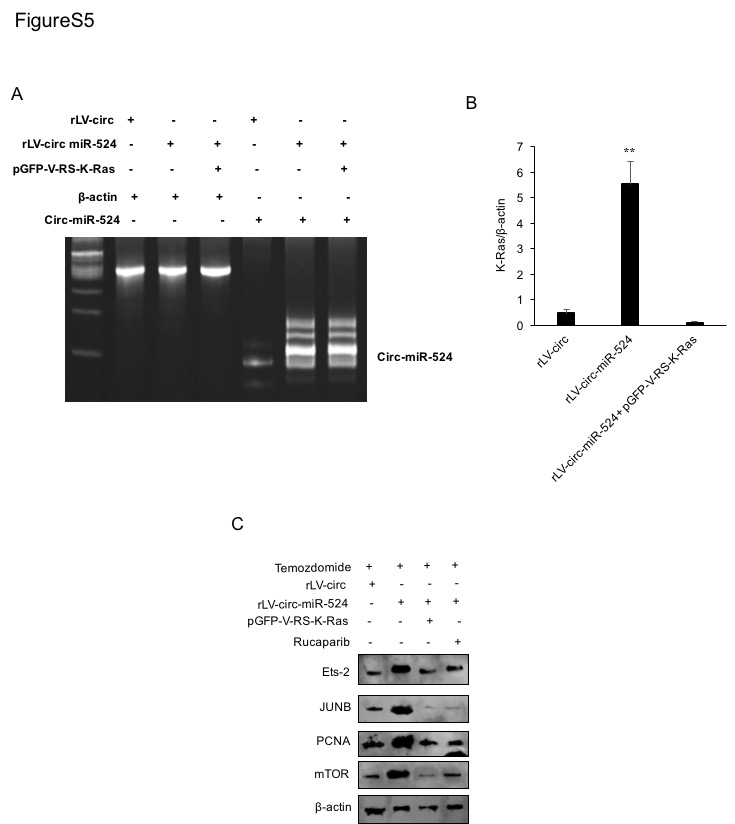


**FigureS5 Circ-miR-524 increases the DNA damage repair ability dependent on K-Ras** A. The Circ-miR-524 was detected by back-to-back reverse transcription polymerase chain reaction (RT-PCR). β-actin was used as internal reference gene. B. The translation ability of K-Ras was detected by Western bloting. β -actin was used as the internal reference gene. β -actin is used as internal reference.C. The translational ability of genes were detected by Western bloting. β-actin was used as the internal reference gene.


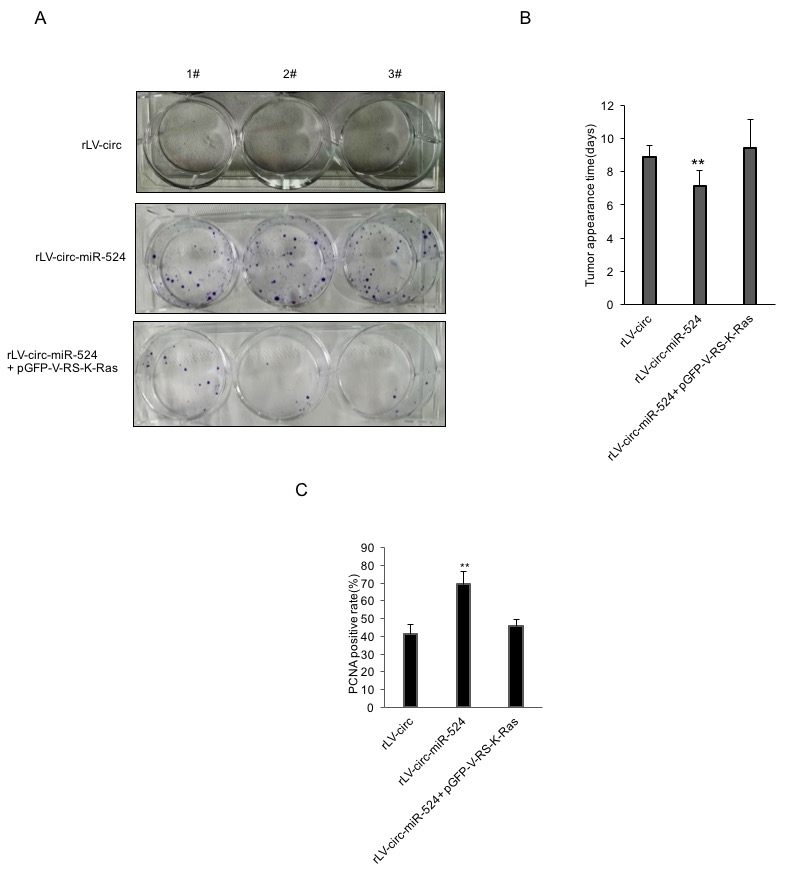


**FigureS6 K-RAS determines the carcinogenic function of Circ-miR-524**  A. The colony forming ability of cells was measured. The photos of plate colonies. B. Comparison of appearance time of xenograft tumor. The values of each group were expressed as mean ± SD (n = 7), * *, P < 0.01, and *, P < 0.05. C. Comparison of PCNA positive rate(%). The values of each group were expressed as mean ± SD (n =7), * *, P < 0.01, and *, P < 0.05.

**Discussion**

In this study, we clearly demonstrate that Circ-miR-524 alter transcriptome and proteome, and accelerates the growth of liver cancer cells.  To our knowledge, this is the first report demonstrating Circ-miR-524 function in liver malignant progression. Studies are now indicated to evaluate the effects in experimental models and illustrates the mechanisms for Circ-miR-524 promoting malignant growth of liver cancer. These results provide basis for human liver cancer prevention, diagnosis and treatment.

It is obviously good that Circ-miR-524 promotes the proliferation of liver cancer cells *in vitro and in vivo* and possesses strong oncogenic action*.* Previous research has demonstrated the involvement of circRNAs in tumorigenesis. CircRNA_101996 promotes growth of human cervical cancer cells by upregulating TPX2（1）. Circ ASAP1 regulates the expression of MAPK1 to promote proliferation of liver cancer cells （2）. However, the role and molecular mechanism of Circ-miR-524 in hepatocarcinogenesis has been poorly elucidated. Further investigation is needed to speculate how Circ-miR-524 functions through mechanisms similar to those of aforementioned circRNAs.

It is worth mentioning that Circ-miR-524 effect on transcriptome in liver cancer. For examples, Circ-miR-524 up-regulated genes, including HSPA8P5,MATR3, SUMO2P21, SLC38A6, PTENP1, CDC25A,VPS13A,SLC7A5P1,MSANTD3,MSANTD3-TMEFF1, PRSS23,LSM8, E2F5,NPM1P39, CCDC83, RPS23, ISY1-RAB43. Circ-miR-524 down-regulated genes , including MYO6, FTH1P5, FAM83C, UPB1, HERPUD1, CLCN6,EIF5AL1, ENO2,CELSR3,PRRT1,CCN1. UBE2M promotes cell proliferation via the β-catenin/cyclin D1**(3)**.CHMP2A inhibits apoptosis (**4,5**).SLC6A6 regulates Wnt/β-catenin signaling pathway(**6**,**7**).Muscle-specific Cand2 is upregulated by mTORC1 (**8**).The P3H4 functions an oncogene(**9**).MKRN2 promoted ubiquitination-mediated degradation of PKM2 (**10)**. CISD3 inhibition induced ferroptosis**(11**). BCAT1 fuels branched-chain ketoacid in tumours(**12)** .We speculate that these genes play an important role in the carcinogenic function of Circ-miR524, but further research is needed.

On the other hand, it is obvious that Circ-miR-524 alter proteome in liver cancer . For examples, Circ-miR-524 up-regulated genes , including FN1,UBE2M,SEPTIN3,CHMP2A,SMARCE1,SLC6A6,HACL1,RETREG3,CAND2,PRKRA,P3H4, MKRN2, GIPC1, CISD3, TUBB,NISCH,UBE2Z,PLD3,BCAT1,H1-10,. HP1a. MATR3 is involved in DNA repair**(13**). Cdc25 has critical roles in regulating the cell cycle**(14**,**15**).PRSS23 promotes tumor growth of gastric cancer **(16**-**18**). Herpud1 increased the M2 polarization of macrophages (**19)**. ENO2 is a key glycolytic enzyme (**20**).CELSR3 is associated with tumorigenesis (**21**).CCN1 induces autophagy (**22**).Moreover,Circ-miR-524 down-regulates proteins , including UFC1, CLPB,BPNT2,POLR1C,TUBB6,NPC1,PAFAH1B1,CCDC22,RPL21,SET,PARD6G,ISY1-RAB43, SLC6A11,NPC1, PAFAH1B1,LUC7L,SMC4, PRPF4,CTCF,ATG3,GULP1,CARML1,TMEM164,LRP8. Circ-miR-524 up- NT5C3A,RBM2B,MYO6,POFUT1,NEK6,DDX55,L2HGDH,AGO2,HACD3,SPTLC1,ITIH2, MTRF1,H1-10,SMDT1,PPAN-P2RY11, XRCC6,PPP1CC, RABGGTA, TMEM65,ULK1,POFUT1,KRAS,PDK1,TAP1,APAF1,PIK3CB,MAPK1,AK4,FKBP1, SUPV3L1,FKBP2,RBX1,POLR1D,CALR,MLYCD,POLR2H. UFC1 promotes non-small cell lung cancer（NSCLC）progression (**23**). CLPB are a cause of severe congenital neutropenia(**24**). NPC1 inhibits phagocytic uptake (**25**). SET protein is a multi-functional oncoprotein (**26**).Rev-erbα promotes the expressions of Slc6a1 (**27**). Smc4 enhanced NEMO transcription **(28)**.SMC4 enhances malignant biological behaviors of endometrial cancer (**29**). PRP4 promotes skin cancer **(30)**. Cancer-specific CTCF regulates oncogene expression(**31**-**33**). ATG3 contributes to the progression of non-alcoholic fatty liver disease in humans**(34**,**35**).GULP1 regulates KEAP1-NRF2 signaling **(36)**. LRP8 can promote tumorigenesis (**37,38)**. UCA1 promotes malignancy of colorectal Cancer (**39**).POFUT1 activates Notch1 (**40**). POFUT1 can promote tumorigenesis (**41)**.SPOP suppresses AKT kinase activity (**42**).PDK1 promotes ovarian cancer metastasis **(43)**.Hedgehog mediates drug resistance (44).LNC473 enhances APAF1 expression(**45**). PIK3CB regulates AKT signaling pathway **(46**,**47**).MAPK1/3 promotes breast cancer bone metastasis(**48**).The circMAPK1 inhibits the malignant biological behavior of gastric cancer cells (**49)**.SNHG11 upregulates the expression of Twist1**(50)**.Adenylate kinase 4 drives lung adenocarcinoma metastasis(**51)**.RBX1 limits DNA double-strand break repair **52**).POLR1D promotes colorectal cancer progression (**53)**. We speculate that these genes play an important role in the carcinogenic function of Circ-miR-524, but further research is needed.

Circ-miR-524 affects tumor-associated genes in liver cancer cells. Our study identified several important cancer-related genes associated with Circ-miR-524 using high-throughput sequencing methods. Circ-miR-524 regulates some genes expression dependent on K-Ras，e.g. up-regulating Ets-2, CREB-1, mTOR, JUNB, RAF1, GABARAL2, PCNA, FOXD3, PKM2, CDK2, Pim1, EIF5AL1, MYO6, XRCC6, XRCC5, POLR1D, ENO2. Research has shown that ETS2 is overexpressed in colorectal cancer **(54)**. Dysregulation of the mTOR signaling pathway is associated with cancer **(55)**. RAF1 promotes tumor growth **(56)**. PCNA implicated in tumor cell stemness and invasion**(57,58)**. PKM2 promotes cancer progression**(59)**. These genes are hypothesized to play important roles in the oncogenic process mediated by Circ-miR-524, however, the specific mechanisms require further elucidation.

Simultaneously, Circ-miR-524 downregulates the expression of several genes including TP73, PTEN, P18, P19, P57, CUL5, MSH, P21WAF1/CIP1, FAT1, APC, MLH3, LRP8, CDC25A, RPL21, CCDC22, PRSS23, and GADD45. PTEN inhibits tumor progression**(60)**. P18, P19, and P57 exert tumor-suppressive effects**(61,62)**. P62 inhibits proliferation of HCC cells**(63)**. The downregulation of these genes may plays a significant role in the oncogenic process mediated by Circ-miR-524, although the specific mechanisms require further validation.

Our results indicate that K-Ras determines the oncogenic role of Circ-miR-524 in hepatocellular carcinoma. Key evidence includes: 1) Circ-miR-524 promotes the growth ability in vitro and in vivo of human liver cancer cells by enhancing K-Ras expression. 2) Circ-miR-524 regulates some genes expression dependent on K-Ras，e.g. up-regulating Ets-2, CREB-1, mTOR, JUNB, RAF1, GABARAL2, PCNA, FOXD3, PKM2, CDK2, Pim1, EIF5AL1, MYO6, XRCC6, XRCC5, POLR1D, ENO2，down-regulating TP73, PTEN, P18, P19, P57, CUL5, MSH, P21WAF1/CIP1, FAT1, APC, MLH3, LRP8, CDC25A, RPL21, CCDC22, GADD45. 3) Circ-miR-524 increases the binding ability between METTL3 and POLR1D mRNA, METTL3 and JUNB mRNA , and decreases the binding ability between METTL3 and P57 mRNA, METTL3 and APC mRNA, METTL3 and FAT1 mRNA, METTL3 and MLH mRNA. Therefore, increasing the methylation modification ability of POLR1D mRNA、JUNB mRNA and decreasing the methylation modification ability of P57 mRNA, APC mRNA, FAT1 mRNA, MLH mRNA. 4) Circ-miR-524 regulates the K-Ras-protein interaction network in liver cancer cells, the interaction between K-Ras and Ets-2, CREB-1, JUNB, RAF1, PCNA, FOXD3, PKM2, CDK2 were significantly increased, respectively; the interaction between K-Ras and TP73, PTEN, P18, P19, CUL5, P21WAF1/Cip1, P57 were significantly diminished, respectively. 5) Circ-miR-524 enhances the binding ability of H3K36me3 to Rad51, PARP1, ATR, ATM, hMSH6, XRCC5, POLB, SKP2 dependent on K-Ras, which promotes the formation of DNA damage repair complexes and enhances DNA damage repair ability, thereby, increasing the expression of oncogenes Ets-2, JUNB, PCNA, and mTOR in liver cancer cells.

Obviously, these findings are noteworthy that Circ-miR-524 enhances the expression of K-RAS and K-RAS determines the carcinogenic function of miR-Circ-524 in liver cancer. K-RAS is a key clonal oncogenic driver**(64-67)**. Moreover, K-RAS-mutated cancer is associated with MYC degradation and mTOM activiation**(68,69)**. Furthermore, K-RAS causes tumor progression in hepatocellular carcinoma **(70)**. Several miRNAs have been identified to directly inhibit K-Ras, thereby inhibiting tumor progression **(71-73)**. Our results suggest that K-Ras plays a crucial role in the hepatocarcinogenic process mediated by Circ-miR-524, although the detailed mechanisms require further validation.

Furthermore, Circ-miR-524 alters gene expression by promoting DNA damage repair. Circ-miR-524 enhances the binding ability of H3K36me3 to Rad51, PARP1, ATR, ATM, hMSH6, XRCC5, POLB, SKP2 dependent on K-Ras, which promotes the formation of DNA damage repair complexes and enhances DNA damage repair ability, thereby, increasing the expression of oncogenes Ets-2, JUNB, PCNA, and mTOR in liver cancer cells.DNA damage can lead to mutations, cancer **(74)**. DNA mismatch repair maintains genome stability **(75-77).** miR-675 accelerates malignant transformation by inhibiting DNA mismatch repair **(78)**. MEG3 variant enhances cell DNA damage repair capability **(79)**. Therefore, Circ-miR-524 plays an important role in liver cancer progression by regulating DNA damage repair mechanisms.

*In summary* , the present study depicts a novel provides evidence for Circ-miR-524 to play hepatocarcinogenesis by altering transcriptome, proteome and DNA damage repair. Anyhow, we provides the demonstration that abnormal expression of Circ-miR-524 is very important in hepatocarcinogenesis. Our findings underscore the need for new approaches to further uncover the mechanisms underlying Circ-miR-524-mediated functions in hepatocarcinogensis.

**Reference**

1. T. Song, A. Xu, Z. Zhang, F. Gao, L. Zhao, X. Chen, J. Gao and X. Kong. CircRNA hsa_circRNA_101996 increases cervical cancer proliferation and invasion through activating TPX2 expression by restraining miR-8075. Journal of Cellular Physiology 2019; 234(8): 14296-14305.

2. Z.-Q. Hu, S.-L. Zhou, J. Li, Z.-J. Zhou, P.-C. Wang, H.-Y. Xin, L. Mao, C.-B. Luo, S.-Y. Yu, X.-W. Huang, Y. Cao, J. Fan and J. Zhou. Circular RNA Sequencing Identifies CircASAP1 as a Key Regulator in Hepatocellular Carcinoma Metastasis. Hepatology (Baltimore, Md.) 2020; 72(3): 906-922.

3.Zhang GC, Yu XN, Sun JL, Xiong J, Yang YJ, Jiang XM, Zhu JM. UBE2M promotes cell proliferation via the β-catenin/cyclin D1 signaling in hepatocellular carcinoma. Aging (Albany NY) 2020;12(3):2373-2392

4.Hattori T, Takahashi Y, Chen L, Tang Z, Wills CA, Liang X, Wang HG. Targeting the ESCRT-III component CHMP2A for noncanonical Caspase-8 activation on autophagosomal membranes. Cell Death Differ 2021;28(2):657-670

5.Bernareggi D, Xie Q, Prager BC, Yun J, Cruz LS, Pham TV, Kim W, Lee X, Coffey M, Zalfa C, Azmoon P, Zhu H, Tamayo P, Rich JN, Kaufman DS. CHMP2A regulates tumor sensitivity to natural killer cell-mediated cytotoxicity. Nat Commun2022;13(1):1899

6.Hou X, Wang Z, Ding F, He Y, Wang P, Liu X, Xu F, Wang J, Yang Y. Taurine Transporter Regulates Adipogenic Differentiation of Human Adipose-Derived Stem Cells through Affecting Wnt/β-catenin Signaling Pathway. Int J Biol Sci. 2019;15(5):1104-1112

7.Dazhi W, Jing D, Chunling R, Mi Z, Zhixuan X. Elevated SLC6A6 expression drives tumorigenesis and affects clinical outcomes in gastric cancer. Biomark Med. 2019;13(2):95-104

8.Górska AA, Sandmann C, Riechert E, Hofmann C, Malovrh E, Varma E, Kmietczyk V, Ölschläger J, Jürgensen L, Kamuf-Schenk V, Stroh C, Furkel J. Muscle-specific Cand2 is translationally upregulated by mTORC1 and promotes adverse cardiac remodeling. EMBO Rep. 2021;22(12):e52170

9.Hao L, Pang K, Pang H, Zhang J, Zhang Z, He H, Zhou R, Shi Z, Han C. Knockdown of P3H4 inhibits proliferation and invasion of bladder cancer. Aging (Albany NY). 2020;12(3):2156-2168

10.Liu Z, Xiang S, Guo X, Zhou J, Liao L, Kou J, Zhang J. MKRN2 inhibits the proliferation of gastric cancer by downregulating PKM2. Aging (Albany NY). 2022;14(4):2004-2013

11.Li Y, Wang X, Huang Z, Zhou Y, Xia J, Hu W, Wang X, Du J, Tong X, Wang Y. CISD3 inhibition drives cystine-deprivation induced ferroptosis. Cell Death Dis. 2021;12(9):839

12.Zhu Z, Achreja A, Meurs N, Animasahun O, Owen S, Mittal A, Parikh P, Lo TW, Franco-Barraza J, Shi J, Gunchick V, Sherman MH, Cukierman E. Tumour-reprogrammed stromal BCAT1 fuels branched-chain ketoacid dependency in stromal-rich PDAC tumours. Nat Metab. 2020;2(8):775-792

13.Malik AM, Barmada SJ. Martin 3 in neuromuscular disease: Physiology and pathophysiology. JCI Insight. 2021;6(1):e143948

14.Shen T, Huang S. The role of Cdc25A in the regulation of cell proliferation and apoptosis. Anticancer Agents Med Chem. 2012;12:631–9

15.Wang C, Zeng J, Li LJ, Xue M, He SL. Cdc25A inhibits autophagy-mediated ferroptosis by upregulating ErbB2 through PKM2 dephosphorylation in cervical cancer cells. Cell Death Dis. 2021;12(11):1055

16.Han B, Yang Y, Chen J, He X, Lv N, Yan R. PRSS23 knockdown inhibits gastric tumorigenesis through EIF2 signaling. Pharmacol Res 2019;142:50-57

17.Karmakar D, Maity J, Mondal P, Shyam Chowdhury P, Sikdar N, Karmakar P, Das C, Sengupta S. E2F5 promotes prostate cancer cell migration and invasion through regulation of TFPI2, MMP-2 and MMP-9. Carcinogenesis 2020;41(12):1767-1780

18.Qi JC, Yang Z, Lin T, Ma L, Wang YX, Zhang Y, Gao CC, Liu KL, Li W, Zhao AN, Shi B, Zhang H, Wang DD, Wang XL, Wen JK, Qu CB. CDK13 upregulation-induced formation of the positive feedback loop among circCDK13, miR-212-5p/miR-449a and E2F5 contributes to prostate carcinogenesis. J Exp Clin Cancer Res. 2021;40(1):2.

19.Li W, Wang Y, Zhu L, Du S, Mao J, Wang Y, Wang S, Bo Q, Tu Y, Yi Q. The P300/XBP1s/Herpud1 axis promotes macrophage M2 polarization and the development of choroidal neovascularization. J Cell Mol Med. 2021;25(14):6709-6720

20.Xu D, Yang F, Fu D, Wang C, Hu B, Zhang Z, Li T, Yan S, Wang X, Nelson PJ, Bruns C, Qin L, Dong Q. Insulin-like growth factor 1-induced enolase 2 deacetylation by HDAC3 promotes metastasis of pancreatic cancer. Signal Transduct Target Ther. 2020;5(1):5

21.Li Y, Zhu L, Hao R, Li Y, Zhao Q, Li S. Systematic expression analysis of the CELSR family reveals the importance of CELSR3 in human lung adenocarcinoma. J Cell Mol Med 2021;25(9):4349-4362

22.Su BC, Hsu PL, Mo FE. CCN1 triggers adaptive autophagy in cardiomyocytes to curb its apoptotic activities. J Cell Commun Signal 2020;14(1):93-100

23.Zang X, Gu J, Zhang J, Shi H, Hou S, Xu X, Chen Y, Zhang Y, Mao F, Qian H, Zhu T, Xu W, Zhang X. Exosome-transmitted lncRNA UFC1 promotes non-small-cell lung cancer progression by EZH2-mediated epigenetic silencing of PTEN expression. Cell Death Dis. 2020;11(4):215

24.Warren JT, Cupo RR, Wattanasirakul P, Spencer DH, Locke AE, Makaryan V, Bolyard AA, Kelley ML, Kingston NL, Shorter J, Bellanné-Chantelot C, Donadieu J, Dale DC, Link DC. Heterozygous variants of CLPB are a cause of severe congenital neutropenia. Blood. 2022;139(5):779-791

25.Colombo A, Dinkel L, Müller SA, Sebastian Monasor L, Schifferer M, Cantuti-Castelvetri L, König J, Vidatic L, Bremova-Ertl T, Lieberman AP. Loss of NPC1 enhances phagocytic uptake and impairs lipid trafficking in microglia. Nat Commun. 2021;12(1):1158

26.Dacol EC, Wang S, Chen Y, Lepique AP. The interaction of SET and protein phosphatase 2A as target for cancer therapy. Biochim Biophys Acta Rev Cancer. 2021;1876(1):188578

27.Zhang T, Yu F, Xu H, Chen M, Chen X, Guo L, Zhou C, Xu Y, Wang F, Yu J, Wu B. Dysregulation of REV-ERBα impairs GABAergic function and promotes epileptic seizures in preclinical models. Nat Commun2021;12(1):1216

28.Wang Q, Wang C, Li N, Liu X, Ren W, Wang Q, Cao X. Condensin Smc4 promotes inflammatory innate immune response by epigenetically enhancing NEMO transcription. J Autoimmun 2018;92:67-76

29.Yan Y, Liu C, Zhang J, Li W, Yin X, Dong L, Pang S, Li X. SMC4 knockdown inhibits malignant biological behaviors of endometrial cancer cells by regulation of FoxO1 activity. Arch Biochem Biophys 2021;712:109026

30.Ahmed MB, Islam SU, Lee YS. PRP4 Promotes Skin Cancer by Inhibiting Production of Melanin, Blocking Influx of Extracellular Calcium, and Remodeling Cell Actin Cytoskeleton. Int J Mol Sci 2021;22(13):6992

31.Kubo N, Ishii H, Xiong X, Bianco S, Meitinger F, Hu R, Hocker JD, Conte M, Gorkin D, Yu M, Li B, Dixon JR, Hu M, Nicodemi M, Zhao H, Ren B. Promoter-proximal CTCF binding promotes distal enhancer-dependent gene activation. Nat Struct Mol Biol 2021;28(2):152-161

32.Fang C, Wang Z, Han C, Safgren SL, Helmin KA, Adelman ER, Serafin V, Basso G, Eagen KP, Gaspar-Maia A, Figueroa ME, Singer BD, Ratan A, Ntziachristos P, Zang C. Cancer-specific CTCF binding facilitates oncogenic transcriptional dysregulation. Genome Biol 2020;21(1):247

33.Song H, Li D, Wang X, Fang E, Yang F, Hu A, Wang J, Guo Y, Liu Y, Li H, Chen Y, Huang K, Zheng L, Tong Q. HNF4A-AS1/hnRNPU/CTCF axis as a therapeutic target for aerobic glycolysis and neuroblastoma progression. J Hematol Oncol 2020 Mar 26;13(1):24

34.da Silva Lima N, Fondevila MF, Nóvoa E, Buqué X, Mercado-Gómez M, Gallet S, González-Rellan MJ, Fernandez U, Loyens A, Garcia-Vence M, Chantada-Vazquez MDP. Inhibition of ATG3 ameliorates liver steatosis by increasing mitochondrial function. J Hepatol2022;76(1):11-24

35.Frudd K, Burgoyne T, Burgoyne JR. Oxidation of Atg3 and Atg7 mediates inhibition of autophagy. Nat Commun 2018;9(1):95

36.Hayashi M, Guida E, Inokawa Y, Goldberg R, Reis LO, Ooki A, Pilli M, Sadhukhan P, Woo J, Choi W, Izumchenko E, Gonzalez LM, Marchionni. GULP1 regulates the NRF2-KEAP1 signaling axis in urothelial carcinoma. Sci Signal 2020;13(645):eaba0443

37.Lin CC, Lo MC, Moody R, Jiang H, Harouaka R, Stevers N, Tinsley S, Gasparyan M, Wicha M, Sun D. Targeting LRP8 inhibits breast cancer stem cells in triple-negative breast cancer. Cancer Lett 2018;438:165-173

38.Qiu H, Shen X, Chen B, Chen T, Feng G, Chen S, Feng D, Xu Q. miR-30b-5p inhibits cancer progression and enhances cisplatin sensitivity in lung cancer through targeting LRP8. Apoptosis 2021;26(5-6):261-276

39.Luan Y, Li X, Luan Y, Zhao R, Li Y, Liu L, Hao Y, Oleg Vladimir B, Jia L. Circulating lncRNA UCA1 Promotes Malignancy of Colorectal Cancer via the miR-143/MYO6 Axis. Mol Ther Nucleic Acids 2020;19:790-803

40.Du Y, Li D, Li N, Su C, Yang C, Lin C, Chen M, Wu R, Li X, Hu G. POFUT1 promotes colorectal cancer development through the activation of Notch1 signaling. Cell Death Dis 2018;9(10):995

41.Li D, Lin C, Li N, Du Y, Yang C, Bai Y, Feng Z, Su C, Wu R, Song S, Yan P, Chen M, Jain A, Huang L, Zhang Y, Li X. PLAGL2 and POFUT1 are regulated by an evolutionarily conserved bidirectional promoter and are collaboratively involved in colorectal cancer by maintaining stemness. EBioMedicine 2019;45:124-138

42.Jiang Q, Zheng N, Bu L, Zhang X, Zhang X, Wu Y, Su Y, Wang L, Zhang X, Ren S, Dai X, Wu D, Xie W, Wei W, Zhu Y, Guo J. SPOP-mediated ubiquitination and degradation of PDK1 suppresses AKT kinase activity and oncogenic functions. Mol Cancer 2021;20(1):100

43.Siu MKY, Jiang YX, Wang JJ, Leung THY, Ngu SF, Cheung ANY, Ngan HYS, Chan KKL. PDK1 promotes ovarian cancer metastasis by modulating tumor-mesothelial adhesion, invasion, and angiogenesis via α5β1 integrin and JNK/IL-8 signaling. Oncogenesis 2020;9(2):24

44.Zhou XT, Ding J, Li HY, Zuo JL, Ge SY, Jia HL, Wu J. Hedgehog signalling mediates drug resistance through targeting TAP1 in hepatocellular carcinoma. J Cell Mol Med 2020;24(7):4298-4311

45.Wu H, Hu X, Li Y, Chen Q, Sun T, Qiao Y, Qin W, Wu Z, Fu B, Zhao H, Zhang R, Wei M. LNC473 Regulating APAF1 IRES-Dependent Translation via Competitive Sponging miR574 and miR15b: Implications in Colorectal Cancer. Mol Ther Nucleic Acids 2020;21:764-779

46.Tian J, Zhu Y, Rao M, Cai Y, Lu Z, Zou D, Peng X, Ying P, Zhang M, Niu S, Li Y, Zhong R, Chang J, Miao X. N6-methyladenosine mRNA methylation of PIK3CB regulates AKT signalling to promote PTEN-deficient pancreatic cancer progression. Gut. 2020;69(12):2180-2192

47.Qu J, Zheng B, Ohuchida K, Feng H, Chong SJF, Zhang X, Liang R, Liu Z, Shirahane K, Mizumoto K, Gong P, Nakamura M. PIK3CB is involved in metastasis through the regulation of cell adhesion to collagen I in pancreatic cancer. J Adv Res. 2021;33:127-140

48.Deng R, Zhang HL, Huang JH, Cai RZ, Wang Y, Chen YH, Hu BX, Ye ZP, Li ZL, Mai J, Huang Y, Li X, Peng XD, Feng GK, Li JD, Tang J, Zhu XF. MAPK1/3 kinase-dependent ULK1 degradation attenuates mitophagy and promotes breast cancer bone metastasis. Autophagy. 2021;17(10):3011-3029

49.Jiang T, Xia Y, Lv J, Li B, Li Y, Wang S, Xuan Z, Xie L, Qiu S, He Z, Wang L, Xu Z. A novel protein encoded by circMAPK1 inhibits progression of gastric cancer by suppressing activation of MAPK signaling. Mol Cancer. 2021;20(1):66

50.Xu L, Huan L, Guo T, Wu Y, Liu Y, Wang Q, Huang S, Xu Y, Liang L, He X. LncRNA SNHG11 facilitates tumor metastasis by interacting with and stabilizing HIF-1α. Oncogene. 2020;39(46):7005-7018

51.Jan YH, Lai TC, Yang CJ, Lin YF, Huang MS, Hsiao M. Adenylate kinase 4 modulates oxidative stress and stabilizes HIF-1α to drive lung adenocarcinoma metastasis. J Hematol Oncol. 2019;12(1):12

52.Xie Y, Liu YK, Guo ZP, Guan H, Liu XD, Xie DF, Jiang YG, Ma T, Zhou PK. RBX1 prompts degradation of EXO1 to limit the homologous recombination pathway of DNA double-strand break repair in G1 phase. Cell Death Differ. 2020;27(4):1383-1397

53.Wang M, Niu W, Hu R, Wang Y, Liu Y, Liu L, Zhong J, Zhang C, You H, Zhang J, Lu L, Wei L, Xiao W. POLR1D promotes colorectal cancer progression and predicts poor prognosis of patients. Mol Carcinog. 2019;58(5):735-748

54. Y. Chen, Y. Ying, M. Wang, C. Ma, M. Jia, L. Shi, S. Wang, X. Zheng, W. Chen and X.-S. Shu. A distal super-enhancer activates oncogenic ETS2 via recruiting MECOM in inflammatory bowel disease and colorectal cancer. Cell Death & Diseases 2023, 14(1): 8.

55. S. Huang. mTOR Signaling in Metabolism and Cancer. Cells, 2020; 9(10):2278

56.Y. Li, M. Pan, T. Lu, D. Yu, C. Liu, Z. Wang and G. Hu. RAF1 promotes lymphatic metastasis of hypopharyngeal carcinoma via regulating LAGE1: an experimental research. Journal of Translational Medicine, 2022; 20(1): 255.

57. A. González-Magaña and F. J. Blanco. Human PCNA Structure, Function and Interactions. Biomolecules 2020;10(4):570

58. Y.-L. Wang, W.-R. Wu, P.-L. Lin, Y.-C. Shen, Y.-Z. Lin, H.-W. Li, K.-W. Hsu and S.-C. Wang. The Functions of PCNA in Tumor Stemness and Invasion. International Journal of Molecular Sciences 2022; 23(10):5679

59.Q. Zhou, Y. Yin, M. Yu, D. Gao, J. Sun, Z. Yang, J. Weng, W. Chen, M. Atyah, Y. Shen, Q. Ye, C.-W. Li, M.-C. Hung, Q. Dong, C. Zhou and N. Ren. GTPBP4 promotes hepatocellular carcinoma progression and metastasis via the PKM2 dependent glucose metabolism. Redox Biology, 2022; 56: 102458.

60. S. Ghafouri-Fard, A. Abak, H. Shoorei, M. Mohaqiq, J. Majidpoor, A. Sayad and M. Taheri. Regulatory role of microRNAs on PTEN signaling. Biomedicine & Pharmacotherapy = Biomedecine & Pharmacotherapie 2021;133: 110986.

61. H. G. Drexler. Review of alterations of the cyclin-dependent kinase inhibitor INK4 family genes p15, p16, p18 and p19 in human leukemia-lymphoma cells. Leukemia, 1998;12(6): 845-859.

62. A. Borriello, I. Caldarelli, D. Bencivenga, M. Criscuolo, V. Cucciolla, A. Tramontano, A. Oliva, S. Perrotta and F. Della Ragione. p57(Kip2) and cancer: time for a critical appraisal. Molecular Cancer Research : MCR 2011; 9(10): 1269-1284.

63. L. Gong, K. Wang, M. Wang, R. Hu, H. Li, D. Gao and M. Lin. CUL5-ASB6 Complex Promotes p62/SQSTM1 Ubiquitination and Degradation to Regulate Cell Proliferation and Autophagy. Frontiers In Cell and Developmental Biology, 2021; 9: 684885.

64. J. L. Bos. ras oncogenes in human cancer: a review. Cancer Research, 1989;49(17): 4682-4689.

65. I. A. Prior, P. D. Lewis and C. Mattos. A comprehensive survey of Ras mutations in cancer. Cancer Res, 2012; 72(10): 2457-67.

66. M. Drosten and M. Barbacid. Targeting the MAPK Pathway in KRAS-Driven Tumors. Cancer Cell, 2020; 37(4): 543-550.

67. V. Amodio, R. Yaeger, P. Arcella, C. Cancelliere, S. Lamba, A. Lorenzato, S. Arena, M. Montone, B. Mussolin, Y. Bian, A. Whaley, M. Pinnelli, Y. R. Murciano-Goroff, E. Vakiani, N. Valeri, W. L. Liao, A. Bhalkikar, S. Thyparambil, H. Y. Zhao, E. de Stanchina, S. Marsoni, S. Siena, A. Bertotti, L. Trusolino, B. T. Li, N. Rosen, F. Di Nicolantonio, A. Bardelli and S. Misale. EGFR Blockade Reverts Resistance to KRAS(G12C) Inhibition in Colorectal Cancer. Cancer Discov 2020; 10(8): 1129-1139.

68. T. K. Hayes, N. F. Neel, C. Hu, P. Gautam, M. Chenard, B. Long, M. Aziz, M. Kassner, K. L. Bryant, M. Pierobon, R. Marayati, S. Kher, S. D. George, M. Long-Term ERK Inhibition in KRAS-Mutant Pancreatic Cancer Is Associated with MYC Degradation and Senescence-like Growth Suppression. Cancer Cell 2016;29(1): 75-89.

69. J. Guo, Y. Liu, J. Lv, B. Zou, Z. Chen, K. Li, J. Feng, Z. Cai, L. Wei, M. Liu and X. Pang. BCL6 confers KRAS-mutant non-small-cell lung cancer resistance to BET inhibitors. J Clin Invest, 2021;131(1):e133090.

70. P. Dietrich, A. Koch, V. Fritz, A. Hartmann, A. K. Bosserhoff and C. Hellerbrand. Wild type Kirsten rat sarcoma is a novel microRNA-622-regulated therapeutic target for hepatocellular carcinoma and contributes to sorafenib resistance. Gut, 2018, 67(7): 1328-1341.

71. X. Chen, X. Guo, H. Zhang, Y. Xiang, J. Chen, Y. Yin, X. Cai, K. Wang, G. Wang, Y. Ba, L. Zhu, J. Wang, R. Yang, Y. Zhang, Z. Ren, K. Zen, J. Zhang and C. Y. Zhang. Role of miR-143 targeting KRAS in colorectal tumorigenesis. Oncogene2009; 28(10): 1385-1392.

72.O. A. Kent, R. R. Chivukula, M. Mullendore, E. A. Wentzel, G. Feldmann, K. H. Lee, S. Liu, S. D. Leach, A. Maitra and J. T. Mendell. Repression of the miR-143/145 cluster by oncogenic Ras initiates a tumor-promoting feed-forward pathway. Genes & Development 2010;24(24): 2754-2759.

73. Y.-D. Luo, X.-Y. Liu, L. Fang, H.-Q. Yu, Y.-J. Zhang, M. Chen, L.-D. Zhang and C.-M. Xie. Mutant Kras and mTOR crosstalk drives hepatocellular carcinoma development via PEG3/STAT3/BEX2 signaling. Theranostics 2022; 12(18): 7903-7919.

74. A. Sancar, L. A. Lindsey-Boltz, K. Unsal-Kaçmaz and S. Linn. Molecular mechanisms of mammalian DNA repair and the DNA damage checkpoints. Annual Review of Biochemistry 2004;73: 39-85.

75. M. Honda, Y. Okuno, S. R. Hengel, J. V. Martín-López, C. P. Cook, R. Amunugama, R. J. Soukup, S. Subramanyam, R. Fishel and M. Spies. Mismatch repair protein hMSH2-hMSH6 recognizes mismatches and forms sliding clamps within a D-loop recombination intermediate. Proceedings of the National Academy of Sciences of the United States of America2014;111(3): E316-E325.

76. D. Liu, G. Keijzers and L. J. Rasmussen. DNA mismatch repair and its many roles in eukaryotic cells. Mutation Research. Reviews In Mutation Research 2017; 773: 174-187.

77. K. C. G. Berg, P. W. Eide, I. A. Eilertsen, B. Johannessen, J. Bruun, S. A. Danielsen, M. Bjørnslett, L. A. Meza-Zepeda, M. Eknæs, G. E. Lind, O. Myklebost, R. I. Skotheim, A. Sveen and R. A. Lothe. Multi-omics of 34 colorectal cancer cell lines - a resource for biomedical studies. Molecular Cancer 2017; 16(1): 116.

78. Y. Lu, S. Song, X. Jiang, Q. Meng, C. Wang, X. Li, Y. Yang, X. Xin, Q. Zheng, L. Wang, H. Pu, X. Gui, T. Li and D. Lu. miR675 Accelerates Malignant Transformation of Mesenchymal Stem Cells by Blocking DNA Mismatch Repair. Mol Ther Nucleic Acids2019; 14: 171-183.

79. Y. Lu, S. Li, S. Song, L. Wang, Y. Chen, X. Jiang, S. Xie, R. Qin and D. Lu. A lncRNA MEG3 variant enhances telomerase activity by increasing DNA damage repair ability in human liver cancer stem cells. Genes & Diseases 2022;10(5):1763-1766

.
